# Modern Contraceptive Use Among Young Women in Kampala Slums: Research Findings from the TOPOWA Study

**DOI:** 10.3390/ijerph22111730

**Published:** 2025-11-15

**Authors:** Monica H. Swahn, Josephine Namuyiga, Gideon Matovu, Charles Natuhamya, Jane Palmier, Anna Nabulya, Harriet Kebirungi

**Affiliations:** 1 School of Public Health, Virginia Commonwealth University, Richmond, VA 23219, USA; 2 Uganda Youth Development Link, Kampala P.O. Box 12659, Uganda; njosephine.1992@gmail.com (J.N.); giram010@gmail.com (G.M.); natuhamyac@gmail.com (C.N.); palmierj@vcu.edu (J.P.); nkanna.kavuma@gmail.com (A.N.); 3 Department of Development Studies, Kyambogo University, Kyambogo, Kampala P.O. Box 1, Uganda; hkebirungi@kyu.ac.ug

**Keywords:** contraceptive use, women’s health, sexual and reproductive health, urban, Kampala, Uganda, Africa

## Abstract

Unintended pregnancies among adolescent and young women in low- and middle-income countries pose major public health challenges, underscoring the need for improved access to modern contraceptives. This study examined prevalence, preferences, and correlates of modern contraceptive use among young women living in urban slums of Kampala, Uganda, to inform targeted interventions. We analyzed baseline data from The Onward Project On Wellbeing and Adversity (TOPOWA), an NIH-funded, multi-component prospective cohort study on mental health among women aged 18–24 years. In 2023, 300 participants were recruited from three sites (Banda, Bwaise, Makindye). Interviewer-administered surveys assessed contraceptive choices, lifestyle, and demographic factors. Modified Poisson regression was used to examine correlates of contraceptive use. Among participants, 66.0% had ever used contraception, 40.0% were current users, and 38.0% reported modern contraceptive use. Multivariable analyses showed that having a consistent partner (PR = 3.28; 95% CI: 1.90–5.67), engaging in sex work (PR = 2.10; 95% CI: 1.46–3.02), older age (PR = 1.08; 95% CI: 1.01–1.16), and having children (PR = 1.72; 95% CI: 1.12–2.66) were associated with higher modern contraceptive use. Findings highlight important gaps in sustained contraceptive use and the need for tailored interventions addressing economic, social, and educational barriers to improve reproductive health in this low-resource setting.

## 1. Introduction

Unintended adolescent pregnancies remain high in low- and middle-income countries (LMICs), with about half ending in unsafe termination [1]. Adolescent fertility and birth rates, however, continue to be elevated in sub-Saharan Africa, Latin America, and Caribbean regions compared to the rest of the world [1,2]. In Africa, the pooled prevalence of adolescent pregnancy is estimated at 19%, being highest in East Africa [3,4], while unintended pregnancy is 30% [5]. Adolescent pregnancies have been associated with adverse outcomes, including eclampsia, fistula, and unsafe abortions, leading to an increase in maternal death [1]. Modern contraceptive use among adolescent girls has been shown to reduce unintended pregnancies and their consequences. However, the prevalence and correlates of preferential use of these methods are not well reported in vulnerable groups and may represent a range of individual, community, and structural factors [3,4,6]. Modern contraceptive methods typically include implants, sterilization, injectables, oral contraceptive pills, intra-uterine device (IUD), condoms, diaphragm, cervical cap, spermicides, Lactational amenorrhea (LAM), vaginal ring, contraception patch, and emergency contraceptive pills [7].

The pooled prevalence of modern contraceptive use is estimated at 24.7% among adolescent girls, 15–19 years, across several countries in Africa [8] but varies substantially by country: 4% in South Sudan, 12.8% in Guinea, 17% in Rwanda, and 52% in Eswatini and Namibia [9,10,11]. In Uganda, the percentage of women of reproductive age using any family planning method is 32.9%, while the percentage using modern contraceptive methods is 29.8% [12], with some estimates suggesting it could be as high as 45% [13]. This prevalence similarly varies significantly by region and population, ranging from 7% in the Karamoja region to 43% in the Eastern region–Kampala at 39% and from 35.2% among women in fishing communities to 61.5% among postpartum teenage mothers in Eastern Uganda [14,15,16,17,18]. Nonetheless, we could not find any study that assesses current modern contraceptive use rates among vulnerable young women in the urban slums of Kampala.

Short-acting contraceptive methods like condoms, oral pills, and injectables are more frequently used than long-acting contraceptive methods like intra-uterine devices (IUDs) and implants in much of Sub-Saharan Africa [15,19,20,21,22,23,24]. The choice of a contraceptive method is influenced by availability and individual knowledge of the available options, which, in some way, is partly determined by their literacy levels. Studies in DRC, Ethiopia, Rwanda, and Burundi showed that women with at least secondary education are more likely to use modern contraception compared to women with lower education [25,26,27,28]. Similarly, in Uganda, married women are more likely to use modern contraception if they or their partners have completed at least secondary education [13,20]. The fear of side effects that comes with using certain methods is another factor that negatively influences modern contraceptive use. A study conducted in Blantyre–Malawi found that women who feared side effects were 3 times more likely to report the unmet need for modern contraception [29]. Similar concerns have been reported in Zambia [30], Nigeria [31], and Uganda [14]. Urban young women living in poverty in Kampala slums are often unable to afford and access education, contraceptive information, or quality healthcare service [32], yet little is known about their modern contraceptive use.

Socio-cultural norms in many settings across sub–Saharan Africa also bar adolescents from accessing modern contraceptive services. A qualitative study conducted in Uganda revealed that parents and guardians perceived the modern contraceptive use by adolescents as promoting sexual promiscuity and as unacceptable to cultural, religious, and moral norms [33]. This is in line with other findings in Kenya [34] and Uganda [35]. Further, religious affiliation, such as identifying as Muslim, has been associated with lower levels of modern contraceptive use [8,13,36]. In Kenya, gender-based violence and family conflicts have been cited as consequences of covert modern contraceptive use [37], and the repercussions, as predicted, included inconsistencies, unreliability, lack of social and financial support, and social sanctioning as female disobedience [38,39]. Urban vulnerable adolescents and young women are not exempt from these hardships in accessing modern contraceptives, yet remain understudied.

Engaging in multiple sexual encounters, as with transactional sex or married/cohabiting couples, increases the likelihood of modern contraceptive use [40,41]. However, in contrast, adolescents engaged in such sexual relationships often suffer intimate partner violence (IPV) [42], which lessens their probability of using modern contraception when they want to [29,37,43]. While vulnerable young women living in urban slums are predisposed to sexual activity and IPV earlier in life [44,45], modern contraceptive use in this population is still unclear.

Among other factors that influence modern contraceptive use is access to sexual reproductive health information and services. Lack of youth-friendly reproductive health services at public facilities is a major barrier to modern contraceptive use, prompting young women to seek services from private healthcare providers who may be less equipped [37,46,47,48]. A study in Uganda revealed that 40% of healthcare facilities in informal settlements offered contraceptives, mostly privately owned. Only a third provided at least one long-acting method, and 25% did not offer contraceptives to unmarried adolescents [49]. Incentivized modern contraceptive use has been shown to significantly increase family planning uptake among poor urban communities; however, this has not yet been implemented widely [50]. Additionally, women exposed to mass media such as radio and TV showed higher levels of modern contraceptive use [21,31].

There is a dearth of knowledge on the prevalence and preference of modern contraceptive use and the associated factors among vulnerable young women living in urban slums of Kampala, Uganda. To understand the factors influencing young women’s choice of contraception, it is essential to consider individual, social, community, and structural factors that may affect their access to modern contraceptive methods. This is particularly the case for young women living in poverty, where there are high levels of stress and depressive symptoms [51], where access to healthcare and education can be limited, and where survival sex and IPV levels tend to be high [44,52]. It is within this context that this current study sought to address the research question: What is the prevalence, preference, and correlates of modern contraceptive use among young women living in poverty in Kampala, Uganda? We were particularly interested in examining those factors well established in previous research, such as demographic characteristics, as well as other psychosocial health factors, such as HIV testing, having a partner, and engaging in sex work. However, we also wanted to determine if current alcohol use, IPV, and psychological distress may be associated with modern contraceptive use. Our previous research with young women in Kampala indicated that alcohol use during sex was strongly associated with pregnancy [32]. Similarly, IPV and associated distress also seem to impact contraceptive use [29,37,43]. As such, we will include these factors in our analysis to determine if they are associated with modern contraceptive use. By examining these diverse factors, we can better identify the barriers and facilitators to modern contraceptive use in this population and develop targeted interventions to improve access and informed reproductive choices.

## 2. Methods

### 2.1. Study Participants

Between July 2023 and November 2023, we enrolled 300 young women to participate in a prospective observational multi-component cohort study across three sites in Kampala (i.e., Banda, Bwaise, and Makindye) to examine the mechanistic pathways of mental illness (TOPOWA study). Details of the study have been described previously [53,54]. The target population for the TOPOWA cohort study was those aged 18 to 24 who self-reported as female, lived within a radius of 2 km from the Uganda Youth Development Link (UYDEL) vocational training centers, and had attained a minimum of primary five education level. Those who self-reported current pregnancy, had significant intellectual disability, severe mental illness, or substance use requiring hospitalization were excluded from the study. Purposive sampling was used to recruit participants, guided by local leaders until the sample size was reached. Among the 495 young women screened, 137 were not eligible for participation, and 58 did not turn up for study enrollment, making the participation rate 83% among those eligible. In this paper, we present analyses using the baseline data from the TOPOWA cohort study.

As part of the baseline assessment, participants were asked to complete an interviewer-administered survey containing a broad range of measures pertaining to demographic and psychosocial characteristics and life experiences, in addition to other study components. The study was conducted in accordance with the ethical declaration of Helsinki. It was approved by the Kennesaw State University, Makerere University School of Health Sciences (MaKSHS) Research and Ethics Committee (MAKSHSREC-2023-532) and the Uganda National Council of Science and Technology, UNCST (HS2959ES). All participants provided written informed consent before taking part in the study. Participants also received remuneration for participating in the survey and the other data collection protocol components.

### 2.2. Measures

#### 2.2.1. Contraceptive Use

During the baseline assessment, women were asked, “Have you ever used any contraceptives?” to measure lifetime contraceptive use. To assess current contraceptive use, women were asked, “What is your current method of birth control/family planning?”. Also, responses to the question were coded to form a binary variable: 0 (non-user or traditional contraceptive user) and 1 (modern contraceptive user) to determine the prevalence and women’s characteristics associated with modern contraceptive use. Modern contraceptive methods include implants, sterilization, injectables, oral contraceptive pills, intra-uterine device (IUD), condoms, diaphragm, cervical cap, spermicides, Lactational amenorrhea (LAM; infant less than 6 months old, menstruation has not returned, and exclusively breast feeding), vaginal ring, contraception patch, emergency contraceptive pills and traditional methods include withdrawal, fertility awareness, and abstinence [7]. In addition, women were asked, “What is the reason for using this current method of birth control/family planning?” to assess the contraceptive preferences.

#### 2.2.2. Demographic Characteristics

The survey also collected information that included age, education level, the living status of participants’ parents, and parenting status, among others. During analysis, we only included demographic characteristics relevant to the study objective.

To assess the women’s living standards, the wealth index—a routine measure of socioeconomic inequality [55]—was used in this study. The wealth index measure was derived from information on household assets and characteristics (e.g., television, bicycles, cows, and poultry) collected using the baseline financial stressors survey. Factor analysis, one of the methods for constructing the wealth index [56], was applied, and data were all coded as binary indicator variables.

Inequality in modern contraceptive use was assessed using (i) wealth categorized as low and high, (ii) level of education attained (primary or lower, some secondary or higher), and other characteristics.

#### 2.2.3. Psychological Distress

A later version (K6) [57] of the Kessler Psychological Distress Scale (K10) [58] was used to assess distress in this study. The scale collected information on respondents’ past month’s experience of six symptoms: nervousness, hopelessness, restlessness or fidgetiness, severe depression, difficulty, and worthlessness. The responses were rated as 0 (none of the time), 1 (a little of the time), 2 (some of the time), 3 (most of the time), and 4 (all of the time). The sum score was obtained by summing up all scores, excluding items with missing values. The score ranged from 0 to 24, and a higher score indicated higher psychological distress. A score of 13 or higher suggested nonspecific severe psychological distress [59]. The scale’s internal consistency was high (Cronbach’s α = 0.80).

#### 2.2.4. Intimate Partner Violence (IPV)

We used 13 items of the WHO violence against women tool [60] with three constructs: psychological, physical, and sexual violence. Respondents were asked whether their current husband/partner, or any other partner, ever insulted or made them feel bad about themselves, physically forced them to have sexual intercourse when they did not want to. Responses were “yes” or “no”. In this study, a woman was categorized as having ever experienced IPV if they agreed to any of the questions.

For the Kessler Psychological Distress Scale, the adjusted weighted person-mean imputation approach was used to handle any item-level missingness. Since surveys were administered by certified research assistants, missing data was minimal. Overall, only one participant’s item was missing; hence, the missing item was replaced using a weighted person-mean imputation method, ensuring that individual scores accurately reflected the intended scale total structure while maintaining the reliability of the data.

### 2.3. Statistical Analysis

Demographic characteristics, used to describe the study population, were presented by frequencies and percentages, while the prevalence of modern contraceptive use was presented using proportions by categories of demographic characteristics and other variables deemed to be associated with modern contraceptive use according to previous studies.

To assess inequality in modern contraceptive use by wealth and education level, we used predicted probabilities derived from regression models for binary outcomes [61]. For example, to assess the impact of education on modern contraceptive use, we compared the expected probabilities of modern contraceptive use for a woman with primary or lower education and a woman with some secondary or higher education who were the same age. Women’s age, education, and socioeconomic status (measured by wealth index) were considered during this analysis as they are common predictors of modern contraceptive use in Uganda [62,63] and other sub-Saharan African countries [64].

A modified Poisson model approach, useful for estimating relative risk by combining a log Poisson regression model with robust variance estimation [65], was applied to assess factors associated with modern contraceptive use. The primary outcome was modern contraceptive use coded 1 (current modern contraceptive users) and 0 (otherwise). Also, a consistent sex partner was defined as any person the participant regularly engaged in sexual intercourse with: boyfriend/husband/sex client. Instead of the odds ratio, we estimated the prevalence ratio to measure the associations since the prevalence of the study outcome was greater than 10% (common outcome); hence, the odds ratios would have considerably overestimated the strength of the association [66]. To identify factors associated with modern contraceptive use, variables with strong theoretical importance and those that were significant at bivariate analysis were added to the multivariable regression model. Multicollinearity was tested using Variance Inflation Factors (VIFs) at a cut point of 10 [67]. Although the VIF for age was slightly above 10, it was retained due to its strong theoretical importance as a primary predictor of contraceptive use. In addition, comparing standard errors across models with and without age revealed negligible differences, indicating that the level of multicollinearity for age was tolerable [68].

Stata 15.0 (StataCorp, College Station, TX, USA) was used for all analyses, and the statistical significance was set a priori at *p* < 0.05.

## 3. Results

### 3.1. Demographic Characteristics

Of the 300 women study participants, most had attained some secondary or higher level of education (66.0%) and had children (62.0%). The majority of the women did not live together with their parents (66.3%) and had ever tested for HIV (90.3%). Details of these results are presented in Table 1.

### 3.2. Contraceptive Use Prevalence and Methods

In terms of contraceptive use, 198 of the 300 women (66.0%) had ever used any contraceptive method in their lifetime. The prevalence of overall current contraceptive use was 40.0%, while that of current modern contraceptive use was 38.0%.

The prevalence of current modern contraceptive use by reproductive health characteristics, wealth index, IPV, psychological distress, past-month alcohol use, and demographic characteristics is presented in Table 2. The overall current modern contraceptive use was 38.0% (95% CI: 32.7–43.6). The current modern contraceptive use varied significantly by having a consistent partner, age, having children, engaging in sex work, past-month drinking, IPV, HIV testing, household size, and living with parents. Modern contraceptive use was highest among women with consistent partners 51.8% (95% CI: 44.8–58.7), who were between 21 and 24 years of age 49.4% (95% CI: 41.6–57.2), who had children 51.6% (95% CI: 44.4–58.7), and who were engaged in sex work 90.9% (95% CI: 55.9–98.7). Modern contraceptive use was also more prevalent among women who drank alcohol in the past month 54.4% (95% CI: 42.5–65.8), had experienced any IPV 47.3% (95% CI: 39.8–53.2), had ever tested for HIV 41.3% (95% CI: 35.6–47.3), were living in households with four or fewer members 47.9% (95% CI: 40.5–55.5) and were not living with their parents 47.9% (95% CI: 40.9–55.0).

**Figure 1:**
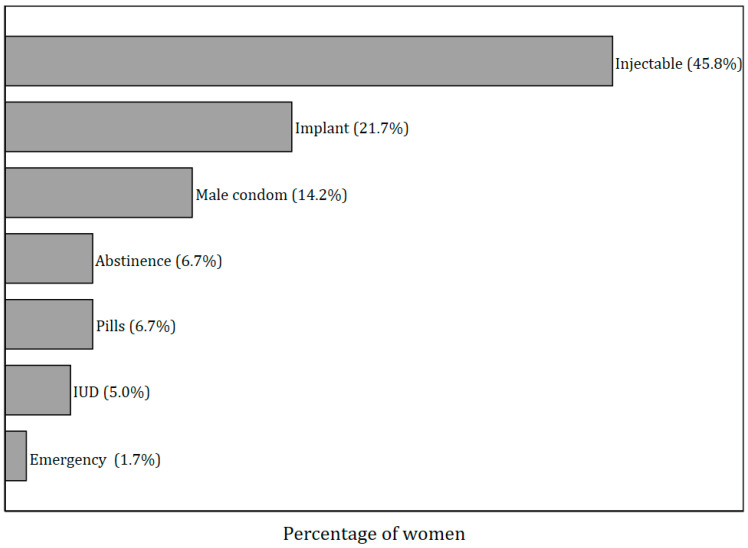
Contraceptive use methods at the baseline assessment of the TOPOWA study among women who reported any current contraceptive use (*n* = 120).

**Figure 2:**
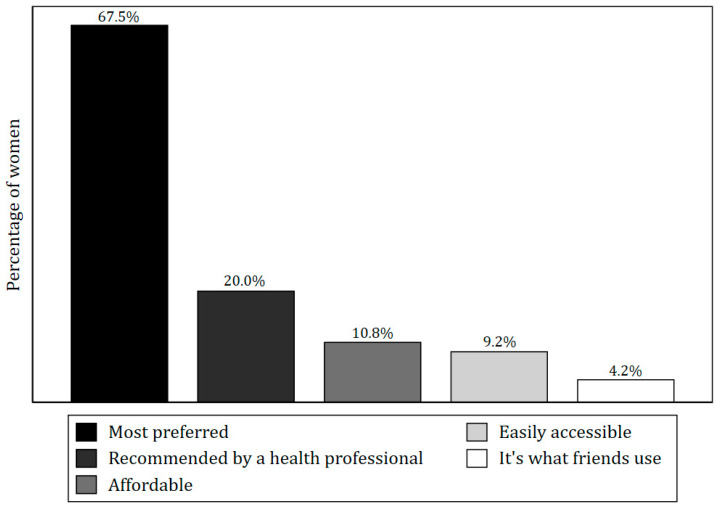
Reasons for using the contraceptive method as reported by women in the TOPOWA baseline assessment who were currently using any contraceptives (*n* = 120).

The most frequently reported contraceptive method was injectables, as reported by 45.8% of all current contraceptive users, followed by implants (21.7%), while the least popular method was emergency contraceptive pills (1.7%) (Figure 1). The predominant reason for the choice of the current contraceptive method was user preference (67.5%) as indicated in Figure 2.

### 3.3. Modern Contraceptive Use Inequality by Wealth and Education Level

**Figure 3:**
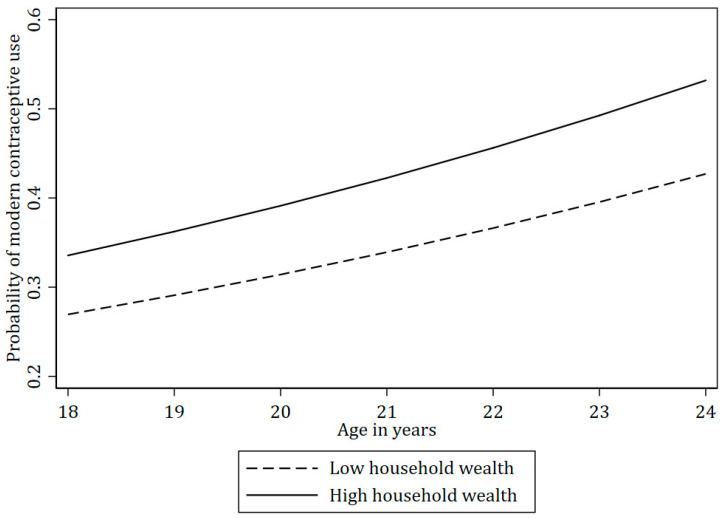
The probability of modern contraceptive use by household wealth index across age in the TOPOWA Baseline Assessment (N = 300).

**Figure 4:**
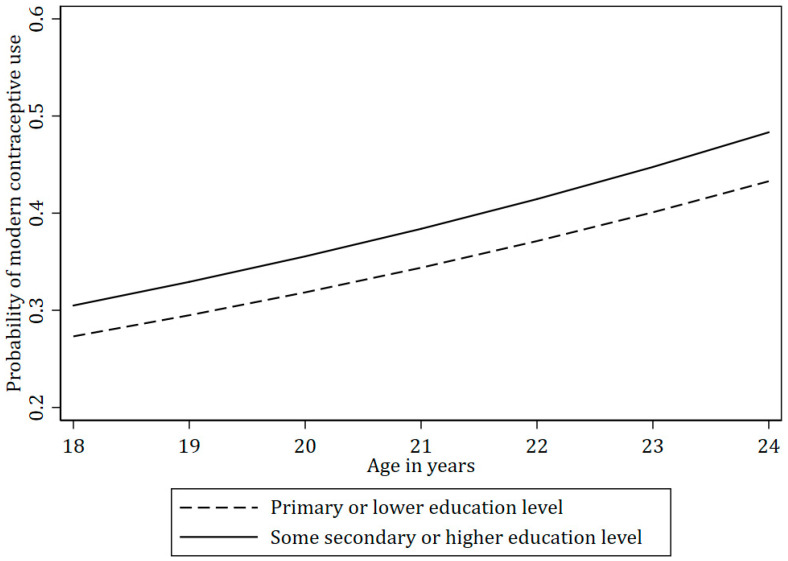
The probability of modern contraceptive use by education level across age among women in the TOPOWA study (N = 300).

The expected probability of using modern contraceptives was generally higher among the participants who were 20 years and older (Table 3). Moreover, women from wealthier households were more likely to use contraceptives than those with lower incomes (Figure 3). Women who had attained higher education (i.e., some secondary or higher) were more likely to use contraceptives than those who had primary or lower level of education (Figure 4).

### 3.4. Factors Associated with Modern Contraceptive Use

Results from the multivariable regression model (Table 4) indicate that, holding other variables constant, having a consistent partner was associated with higher modern contraceptive use (Prevalence ratio, PR = 3.28; 95% confidence interval, CI: 1.90–5.67). Engaging in sex work (PR = 2.10; 95% CI: 1.46–3.02), older age (PR = 1.08; 95% CI: 1.01–1.16), and having children (PR = 1.72; 95% CI: 1.12–2.66) were also significantly associated with modern contraceptives use, other variables held constant.

## 4. Discussion

In this study we sought to determine the prevalence and associated characteristics of modern contraceptive use among young women living in poverty in Kampala. Our findings provide valuable insights for planning sexual and reproductive health services for this population to prevent unwanted pregnancies, support family planning, and prevent sexually transmitted diseases.

The overall prevalence of lifetime contraceptive use among the women in the TOPOWA study was relatively high, with 66% having used any contraceptives at some point, comparable to 62% from a study among reproductive-age women in the urban areas of Ethiopia [69]. However, the prevalence of current contraceptive use was considerably lower at 40%, suggesting gaps in ongoing access or sustained use of contraceptives among the women in our study. This finding was slightly higher compared to 33.4% from the Uganda Demographic and Health Survey (UDHS) among the 20–24-year-olds [12] and 32% from a national household survey in a broader age range of 15–24-year-olds in Ghana [22]. Our estimates likely reflect that the women in this study may have increased exposure to contraception knowledge and usage in the urban settings, as reflected in the 2022 UDHS report. The prevalence of modern contraceptive use in our study (38%) is comparable to findings from other studies in sub-Saharan Africa [70,71,72] and much higher in contrast to the 9% among 15–19-year-old females in Uganda’s general population [73], suggesting probable easier access to modern contraception in urban areas than the rural areas. We also found that the most frequently used of the modern methods was injectable contraception, followed by implants, highlighting a probable preference for long-acting reversible contraceptives. This preference aligns with findings from other studies in similar settings [47,70,74,75] where convenience and efficacy of reversible contraceptives drive their popularity. The low use of emergency contraceptives among the study population suggests a potential lack of access to this method, perhaps due to the cost of the contraceptive and other sociocultural factors, or it may reflect a preference for more regular or sustained contraceptive methods to prevent unintended pregnancies [76,77,78]. We did not, however, include questions that enable us to provide additional context or information about the factors that may impact the use of emergency contraceptives.

Our findings also indicate significant disparities in the expected probability of modern contraceptive use based on socioeconomic status. Women with higher living standards in our study, albeit still living in the urban slums, were more likely to use modern contraceptives, which may be attributed to better access to healthcare services, higher health literacy, and perhaps the financial means to afford contraceptives [8,79]. Similarly, women with secondary or higher education were more likely to use modern contraceptives compared to those with less education. Education likely equips women with the knowledge and autonomy to make informed reproductive health decisions, and it may also correlate with greater empowerment and access to resources [25,26,80]. Unemployment and low education levels are often linked to economic hardship and limited access to healthcare, further exacerbating disparities in contraceptive use [63,81].

Our findings also identified several factors significantly associated with modern contraceptive use, including having a consistent partner, sex work, age, and having children. Women with consistent partners were more likely to use modern contraceptives, possibly due to stable relationship dynamics that facilitate discussions about family planning. Also, the women reporting sex work or survival sex were also more likely to report modern contraceptive use, which may also represent an opportunity for integrated care to address both unwanted pregnancies and the prevention of HIV and other sexually transmitted infections [82,83]. Intriguingly and perhaps due to the small sample size, HIV testing was not statistically significant in the multivariable analysis.

Age, even with the narrow range (18–24) of our sample population, also played a significant role, with women over the age of 20 generally more likely to use modern contraceptives. This suggests that younger women may face barriers or lack the necessary knowledge and support for contraceptive use [84]. Moreover, women who are a few years older may be more knowledgeable about contraceptives compared to those slightly younger [15]. Having children was also strongly associated with contraceptive use, likely reflecting a desire to space or limit births. Without statistically controlling for other factors, our findings show that women in smaller households were more likely to use contraceptives, possibly indicating that those with more means live in smaller households and perhaps that household crowding and economic constraints influence family planning decisions [8,72]. Also, IPV of any form was associated with modern contraceptive use only in the bivariate analyses, not in the multivariate models. However, there may be other factors not examined in this study that may influence modern contraceptive use. Previous research has indicated that women may lack autonomy and empowerment regarding their own health issues and decision-making regarding pregnancy, when engaged in a sexual relationship, which reflects relatively common sociocultural norms across African societies [85,86]. This is a key research question for future research, but it would require a larger sample size of women.

The findings from this study have important implications for public health strategies and policies aimed at improving contraceptive access and use among women in low-resource settings. Efforts to promote contraceptive use should focus on increasing accessibility, particularly among younger women, those from lower wealth index, and those who have received less education. Education and community outreach programs that enhance awareness of various contraceptive methods and their benefits could empower women to make informed reproductive health decisions. Moreover, integrating family planning services with other sexual health services may provide a holistic approach to women’s health and encourage contraceptive use, which may be particularly important for women engaged in sex work [87,88]. Additionally, addressing socio-economic barriers by providing subsidized or free contraceptives at private sector clinics could also help bridge the gap in contraceptive use among different groups. Previous research has raised these issues, outlining the importance of socioeconomic status, living in an urban area, age, number of children, and employment in the selection and choice of contraception among adolescent girls and young women [89,90,91].

We also found that past-month alcohol use was associated with contraceptive use in bivariable analyses but not in multivariable analyses. In previous research of young women living in Kampala, alcohol use during sex was the key correlate for pregnancy [32]. Accordingly, alcohol-related condomless sex may be an important factor [92] that should be considered in terms of the prevention of pregnancies and sexually transmitted infections. While the literature regarding alcohol use and sexually transmitted diseases, including HIV [45,93,94], is growing, there is less research on alcohol and its association with contraceptive use and pregnancy, and that is also important to consider for future research. Similarly, IPV and HIV testing were associated with contraceptive use in bivariable analyses but not in adjusted models, suggesting that the effects are largely mediated through relational and structural factors such as partner consistency, sex work, and childbearing, which together shape women’s reproductive autonomy. This is particularly relevant given the intersectional factors impacting these women and the prevalence of survival sex [44] and the intersection of alcohol use, violence, and HIV, which is highly relevant to contraceptive use and pregnancy [45].

There are several limitations that should be considered when interpreting the findings. First, the results presented in this paper are based on cross-sectional baseline data analysis of the TOPOWA cohort study; as such, causal or temporal relationships cannot be inferred. Second, the choice of the current contraceptive method may or may not translate into preference. The relationship between these two factors was not assessed. Further, we did not assess how often or how long the selected method represents the extent of modern contraceptive use. Third, the TOPOWA study excluded women who knew they were pregnant at the time of participant enrollment, likely biasing the findings toward a higher prevalence of contraceptive use among the study participants than in broader population samples, as noted in the comparison with household surveys. Excluding pregnant women was important since other TOPOWA study components required the collection of biomarkers, including saliva samples, which are directly impacted by pregnancy. Moreover, we excluded women who were severely impacted by mental illness or substance use and required hospitalization, given their inability to participate in a community study. The implication of that exclusion on the study findings and contraceptive use specifically is unknown.

Fourth, in analysis, we classify periodic abstinence among traditional methods of contraception, as is the case with UNDESA [7]. This may affect the comparability of our results with other studies that do not classify periodic abstinence as a contraceptive method. Lastly, due to a few numbers of events per predictor, the model might not detect small effects. These factors may have impacted the findings in unknown ways and reduced the generalizability of the study findings to this target population. It is also worth noting that some subgroups, such as women who reported engaging in sex work, had relatively small sample sizes, leading to wide confidence intervals. Hence, such findings should be interpreted with caution.

## 5. Conclusions

This study underscores the importance of understanding the demographic and socio-economic factors influencing contraceptive use among women. Addressing disparities in contraceptive access and use requires targeted interventions that consider socioeconomic status, relationship dynamics, and other contextual factors. By improving access to contraceptives and empowering women with the knowledge and resources to make informed choices, we can enhance reproductive health outcomes and promote greater health equity [95]. These findings contribute to the limited body of evidence on contraceptive use among young women living in urban informal settlements in sub-Saharan Africa, where social drivers, gendered norms, and structural inequities continue to shape reproductive decision-making.

## Figures and Tables

**Table 1:** Women participants’ demographic characteristics in the baseline assessment of the TOPOWA study (N = 300).

Characteristic	Category	*n* (%)
Age	Mean (SD): 20.81 (2.05)	
	≤20	144 (48.00)
	>20	156 (52.00)
Education level	Primary or lower	102 (34.00)
	Some secondary or higher	198 (66.00)
Wealth index	Low	175 (58.33)
	High	125 (41.67)
Has children	No	114 (38.00)
	Yes	186 (62.00)
Household size	≤4	169 (56.33)
	>4	131 (43.67)
Lives with parents	No	190 (63.33)
	Yes	110 (36.67)
Consistent sex partner	No	101 (33.67)
	Yes	199 (66.33)
Engaged in sex work	No	289 (96.33)
	Yes	11 (3.67)
Tested for HIV	No	29 (9.67)
	Yes	271 (90.33)
Past-month alcohol use	No	232 (77.33)
	Yes	68 (22.67)
IPV	No	89 (29.67)
	Yes	211 (70.33)
Psychological distress	No	218 (72.67)
	Yes	82 (27.33)

**Table 2:** Prevalence of modern contraceptive use by women’s characteristics in the baseline assessment of the TOPOWA study (N = 300).

Characteristic	Category	Total	Prevalence (95% CI)	*p*
Overall	-	300	38.0 (32.66–43.65)	-
Age	≤20	144	25.69 (19.20–33.48)	<0.001
	>20	156	49.36 (41.56–57.19)	
Education level	Primary or lower	102	36.27 (27.51–46.05)	0.659
	Some secondary or higher	198	38.89 (32.33–45.88)	
Wealth index	Low	175	38.29 (31.36–45.73)	0.904
	High	125	37.60 (29.53–46.43)	
Has children	No	114	15.79 (10.16–23.71)	<0.001
	Yes	186	51.61 (44.42–58.74)	
Household size	≤4	169	47.93 (40.47–55.48)	<0.001
	>4	131	25.19 (18.47–33.35)	
Lives with parents	No	190	47.89 (40.85–55.02)	<0.001
	Yes	110	20.91 (14.28–29.55)	
Consistent sex partner	No	101	10.89 (6.12–18.64)	<0.001
	Yes	199	51.76 (44.80–58.65)	
Engaged in sex work	No	289	35.99 (30.64–41.71)	<0.001
	Yes	11	90.91 (55.94–98.75)	
Tested for HIV	No	29	6.90 (1.72–23.86)	<0.001
	Yes	271	41.33 (35.59–47.31)	
Past-month alcohol use	No	232	33.19 (27.41–39.53)	0.002
	Yes	68	54.41 (42.50–65.84)	
IPV	No	89	17.98 (11.29–27.40)	<0.001
	Yes	211	46.45 (39.79–53.23)	
Psychological distress	No	218	39.91 (33.59–46.58)	0.267
	Yes	82	32.93 (23.61–43.81)	

CI: confidence interval, *p*: *p*-value.

**Table 3:** Modern contraceptive use by marginal probability estimates of wealth index and education levels by age in the baseline assessment of the TOPOWA study (N = 300).

Age	Wealth		Education	
	Group	Marginal *p* (95% CI)	Level	Marginal *p* (95% CI)
18	Low	0.27 (0.19–0.35)	Primary or lower	0.27 (0.19–0.36)
	High	0.34 (0.23–0.44)	Some secondary or higher	0.30 (0.21–0.40)
19	Low	0.29 (0.22–0.37)	Primary or lower	0.29 (0.21–0.38)
	High	0.36 (0.27–0.45)	Some secondary or higher	0.33 (0.25–0.41)
20	Low	0.31 (0.25–0.38)	Primary or lower	0.32 (0.24–0.40)
	High	0.39 (0.31–0.47)	Some secondary or higher	0.36 (0.29–0.42)
21	Low	0.34 (0.28–0.40)	Primary or lower	0.34 (0.26–0.42)
	High	0.42 (0.34–0.50)	Some secondary or higher	0.38 (0.32–0.44)
22	Low	0.37 (0.30–0.43)	Primary or lower	0.37 (0.28–0.46)
	High	0.46 (0.37–0.54)	Some secondary or higher	0.41 (0.35–0.47)
23	Low	0.40 (0.32–0.47)	Primary or lower	0.40 (0.29–0.51)
	High	0.49 (0.39–0.60)	Some secondary or higher	0.45 (0.37–0.52)
24	Low	0.43 (0.33–0.53)	Primary or lower	0.43 (0.30–0.57)
	High	0.53 (0.39–0.67)	Some secondary or higher	0.48 (0.38–0.58)

CI: confidence interval, *p*: probability.

**Table 4:** Adjusted prevalence ratios for the association between modern contraceptive use and women’s characteristics in the baseline assessment of the TOPOWA study (N = 300).

Characteristic	Category	Bivariate	Multivariable
		CPR (95% CI)	APR (95% CI)
Age	-	1.18 (1.10, 1.26) ***	1.08 (1.01, 1.16) *
Education level	Primary or lower	Ref.	Ref.
	Some secondary or higher	1.07 (0.79, 1.46)	1.12 (0.84, 1.48)
Wealth index	Low	Ref.	Ref.
	High	0.98 (0.73, 1.32)	1.25 (0.96, 1.62)
Has children	No	Ref.	Ref.
	Yes	3.27 (2.09, 5.11) ***	1.75 (1.12, 2.74) *
Household size	≤4	Ref.	Ref.
	>4	0.53 (0.38, 0.73) ***	0.78 (0.56, 1.08)
Lives with parents	No	Ref.	Ref.
	Yes	0.44 (0.29, 0.65) ***	0.76 (0.52, 1.13)
Consistent sex partner	No	Ref.	Ref.
	Yes	4.75 (2.67, 8.44) ***	3.20 (1.83, 5.59) ***
Engaged in sex work	No	Ref.	Ref.
	Yes	2.53 (1.98, 3.22) ***	2.24 (1.55, 3.23) ***
Tested for HIV	No	Ref.	Ref.
	Yes	5.99 (1.56, 23.05) **	2.08 (0.56, 7.77)
Past-month alcohol use	No	Ref.	Ref.
	Yes	1.64 (1.23, 2.18) **	1.19 (0.91, 1.57)
IPV	No	Ref.	Ref.
	Yes	2.58 (1.62, 4.12) ***	1.28 (0.85, 1.94)
Psychological distress	No	Ref.	-
	Yes	0.83 (0.58, 1.17)	-

CPR: crude prevalence ratio, APR: adjusted prevalence ratio, CI: confidence interval, Ref.: reference category. * *p* < 0.05, ** *p* < 0.01, *** *p* < 0.001.

## Data Availability

The data presented in this study will become available upon the completion of the cohort study.

## Abbreviations

The following abbreviations are used in this manuscript:

CI	Confidence Interval
HIV	Human Immunodeficiency Virus
IPV	Intimate Partner Violence
IUD	Intra-Uterine Device
MaKSHS	Makerere University School of Health Sciences
LAM	Lactation Amenorrhea Method
LMICs	Low- and Middle-Income Countries
PR	Prevalence Ratio
TOPOWA	The Onward Project On Wellbeing and Adversity
UYDEL	Uganda Youth Development Link
